# Modeling and Control of Colorectal Cancer

**DOI:** 10.1371/journal.pone.0161349

**Published:** 2016-08-18

**Authors:** Li-Peng Song, Hao-Yu Wang

**Affiliations:** Department of Computer Science and Technology, North University of China, Taiyuan, Shanxi, China; Shanxi University, CHINA

## Abstract

Colorectal Cancer (CRC) is becoming a major threat to people’s life in China. Screening methods adopted by many other countries as effective counter-cancer methods have not been explicitly explored for people there. Thus, we present a Markov model with detailed precancerous adenoma states and then evaluate various screening strategies in this paper. Different from current researches, our model considers the population’s heterogeneous risk of developing adenomas and observation-based screening strategies. Furthermore, we also give a new cost-effectiveness metric. After calibrating, the model is simulated using the Monte Carlo method. Numerical results show that there are threshold values of compliance rates below which strategy with every ten-year colonoscopy becomes the most cost-effective method; otherwise, an observation-based screening strategy is the most cost-effective. We also find that strategy with single colonoscopy for adenoma-free individuals and every three-year colonoscopy for those with adenoma is recommended when the observation-based strategy is not considered. Our findings give an explicit and complete instruction in CRC screening protocol in average-risk Chinese.

## Introduction

Colorectal Cancer (CRC) takes the second and forth position in incidence and mortality respectively in China [1]. To make matters worse, its incidence and mortality continue to increase in the country [2]. CRC can often be cured if it is detected at the early stages [3]. Nevertheless, CRC has no symptoms in its early stages. Thus, many advanced screening technologies and strategies have been presented, which can reduce CRC incidence and mortality by detecting and removing adenomas before cancer.

All screening technologies, including guaiac faecal occult blood test (gFOBT), faecal immunochemical test(FIT), DNA, colonoscopy and so on [4], can be divided into two classes: non-invasive stool and blood tests, invasive imaging techniques. These screening methods differ from each other in their screening programs. Non-invasive tests are less expensive but with lower sensitivity to adenoma. Invasive techniques, however, have a higher sensitivity but are more expensive and with a risk of serious complications such as bleeding and perforation. Thus, the choice of CRC screening protocols in China should be given based on explicitly cost-effective evaluation of these protocols.

Most countries such as USA, Australia, Europe and some Asian countries have predicated their CRC screening protocols upon conclusions of cost-effectiveness where screening methods, screening interval, age categories, compliance, clinical capacity, sensitivity and so on are considered [4–8]. Using a Markov model to simulate the disease progression through several health states, Wang et al. [9] analyzed the cost-effectiveness of repeat colonoscopy and single colonoscopy for colorectal neoplasm screening in average-risk Chinese. They found that single colonoscopy is a more cost-effective strategy. However, they did not consider the non-invasive screening methods which were recommended as the initial screening step in resource-limited countries [10]. Huang et al [11] further evaluated the cost-effectiveness of both non-invasive and invasive screening technologies and proposed a multi-step screening method for mainland Chinese. Nonetheless, only four health states (normal, polyp, CRC and death) are included in their disease model. In fact, many detailed precancerous adenoma states should be considered to get more precise screening strategies [6].

In this study, we compared the cost-effectiveness of various screening protocols via a Markov model with detailed precancerous adenomas and age-specified state transition probabilities. As FIT plus colonoscopy screening methods show more advantage than corresponding colonoscopy screening methods [10], the basic screening method considered in this paper is FIT plus colonoscopy. We assume that population risks of developing adenomas are heterogeneous. Furthermore, we examined the observation-based screening strategies where the screening interval was adjusted based on the previous screening results and compared them with other fixed-interval screening strategies. To our knowledge, no such research has been done on screening method in average-risk Chinese. Our aim is to give explicit instructions in CRC screening protocols including screening methods, screening interval et al.

The remainder of this paper is organized as follows: Section 2 gives methods used in this paper. In section 3, we describe experiment setting and give numerical simulations. Then, we summarize our work and discuss future work in section 4.

## Methods

The simulation model was established via Chinese population data and published literature. There were also some parameters borrowed from researches in other countries as they were not available in China.

### Natural History Model of CRC

**Fig 1:**
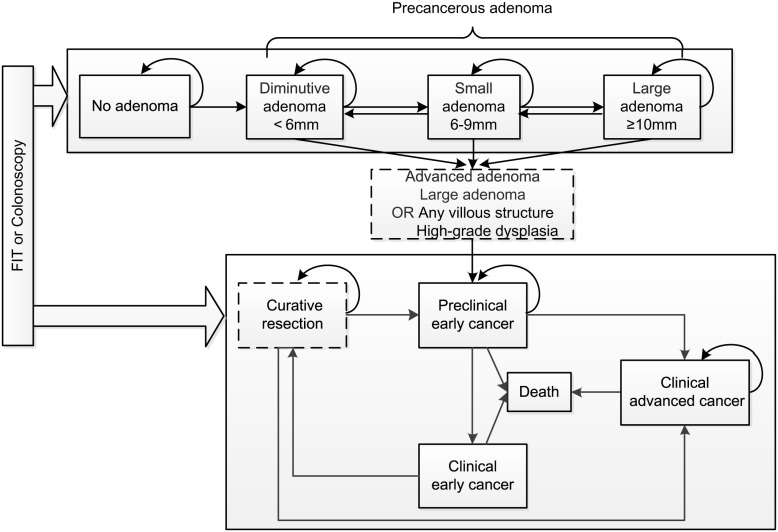
Natural history model of colorectal cancer.

Many mathematical models, such as deterministic differential equation [12–15] and Markov Model, can be used in describing natural history model. Here, we use a discrete-time Markov Model [16] to depict the natural history of CRC. As shown in Fig 1, eight real health states and two virtual states are considered in our model. Individuals change through no adenoma(*N*_*a*_), diminutive adenoma(*D*_*a*_, < 6*mm*), small adenoma(*S*_*a*_, 6–9*mm*), large adenoma(*L*_*a*_, ≥ 10*mm*), preclinical early cancer (*P*_*ec*_), clinical early cancer(*C*_*ec*_), clinical advanced cancer (*C*_*ac*_) and death(*D*_*e*_) states. Some clinical, histopathological or molecular features including size, villosity and dysplasia are considered for each state related to adenoma in the model. Furthermore, state of advanced adenoma and curative resection are virtual states where the former represents the individual with large adenoma or villous structure adenoma and high-grade dysplasia adenoma and the latter represents the patient has curative resection from clinical early cancer state.

We assume that an individual can develop many adenomas at the same time and the maximum of adenomas within an individual is set to ten as in [6]. We also assume that the incidence and progression of new adenomas are independent of other existing adenomas. Moreover, new adenomas are assumed to begin as diminutive and tubular adenomas with low-grade dysplasia. At each time step, adenomas can progress or regress in size and can even become preclinical early cancer. Simultaneously, adenomas can change from tubular to villous and from low-grade to high-grade in villosity and dysplasia, respectively. Preclinical early cancer can become clinical early cancer after screening or deteriorate into clinical advanced cancer or even death. We assume that clinical early cancer can be healed by curative resection but it can also deteriorate into death. However, advanced cancer cannot be cured and individual must receive treatment so that it can maintain the state into advanced cancer until death. Note that only advanced adenoma can progress into preclinical early cancer. Furthermore, both adenoma and cancer can not completely regress, i.e., regression from diminutive adenoma to no adenoma or cancer to any precancerous adenoma.

We assume that the interval time waiting for new adenoma is exponentially distributed. Thus, we can use the Poisson Process to simulate the progression of precancerous adenomas. The individual’s risk of developing adenoma is a baseline risk plus a special risk drawn from the Lognormal Distribution which depicts the population heterogeneity in developing adenomas. The simulation is evolved in discrete time with time horizon *t* = {20, 21, 22, …, 90}. Then, an individual’s health state evolve from age 20 to 90 and the time step is set to one year. Furthermore, we assume that no more than one transition can happen in one time step. Almost all transition rates are age-specified in our simulation model.

### Screening Module

**Fig 2:**
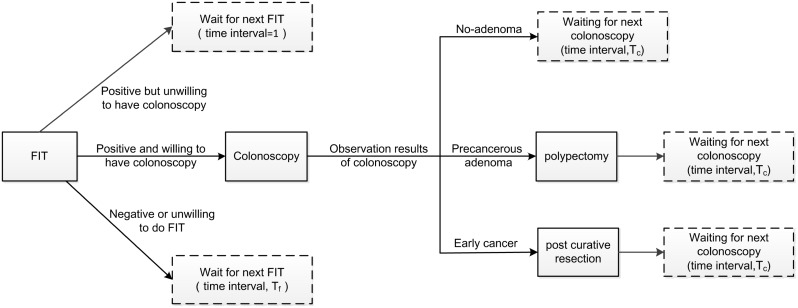
Detailed screening process including FIT and follow-up colonoscopy.

All persons are recommended to take a FIT at the age of initial screening year (*T*_*s*_). Those with a FIT positive result are offered a colonoscopy. Adenomas or cancer, if found, are removed during the colonoscopy or follow-up curative resection, respectively. Then, every a specific time cycle all participants will take part in next colonoscopy. Compliance rates are considered for the initial FIT, 1st and follow-up colonoscopy. Those with a negative FIT result are recommended to take FIT again after each time interval. Moreover, those not compliance with the recommended FIT or colonoscopy are also entering the waiting cycles. The detailed screening process is presented in Fig 2.

As shown in Fig 2, there are two parameters *T*_*f*_ and *T*_*c*_. Here, we concern for these two parameters besides the compliance rates of FIT or colonoscopy. In some screening strategies, *T*_*c*_ is determined based on screening results of previous colonoscopy. Thus, we divide the results into 5 classes: no adenoma, 1-2 diminutive or small adenomas, 3-10 diminutive or small adenomas, advanced adenoma and early cancer. The influences of *T*_*f*_ and *T*_*c*_ are explicitly explored in following experiment section.

### Cost-effectiveness measures

Four indicators including cumulative cost, cumulative quality-adjusted life years (QALYs)[17], incremental cost-effectiveness ratio(ICER) and incremental costs for per CRC prevention (ICPCP) are used to measure the tested screening strategies. Cumulative costs (C) are calculated as below:

$$\begin{array}{c} \left.\begin{array}{ccc} C & = & \sum_{t=T_{s}}^{T_{n}}e^{-\lambda (t-T_{s})}\left(C_{f}I_{f}+C_{c}I_{c}+NC_{p}I_{p}+C_{te}I_{te}+C_{ta}I_{ta}\right), \end{array}\right. \end{array}$$ (1)

where *C*_*f*_, *C*_*c*_, *C*_*p*_, *C*_*te*_ and *C*_*ta*_ represent costs of FIT, colonoscopy and pathology tests, polypectomy of one adenoma, treatment of early cancer and treatment of advanced cancer, respectively; *I*_*f*_, *I*_*c*_, *I*_*p*_, *I*_*te*_ and *I*_*ta*_ denote the probabilities of taking corresponding actions; N is the number of adenomas observed by colonoscopy. T_*s*_ is the starting age of screening, and T_*n*_ is the ending age; *λ* is a discount factor (*λ* = 3%).

QALY is a specific value which depicts a person’s annual utility depending on his health state. Let *U*(*S*) represent the annual utility of a person whose health state is *S*. Then, cumulative QALYs (E) are given as follow:

$$\begin{array}{c} \left.\begin{array}{ccc} E & = & \sum_{t=T_{s}}^{T_{n}}e^{-\lambda (t-T_{s})}U\left(S\right). \end{array}\right. \end{array}$$ (2)

ICER is calculated as (*C*_*A*_−*C*_*B*_)/(*E*_*A*_−*E*_*B*_), where *C*_*A*_ (*C*_*B*_) and *E*_*A*_ (*E*_*B*_) represent cumulative costs and cumulative QALYs of screening method A (B), respectively. Then, a method with lower ICER is a better choice. ICPCP is calculated similarly and also the lower ICPCP the better one method is.

## Model Calibration and Numerical Results

In this section, we first calibrate the Natural History Model of CRC given in previous section. Then, we investigate the effects of screening intervals including the interval between two successive FITs and the interval between two successive colonoscopies. We also investigate the sensitivity of the screening methods to changes in compliance rates of FIT or colonoscopy. In the following sections, we ran each simulation 1000 times for 100,000 asymptomatic individuals aged 20 years.

### Natural History Model Calibration

**Fig 3:**
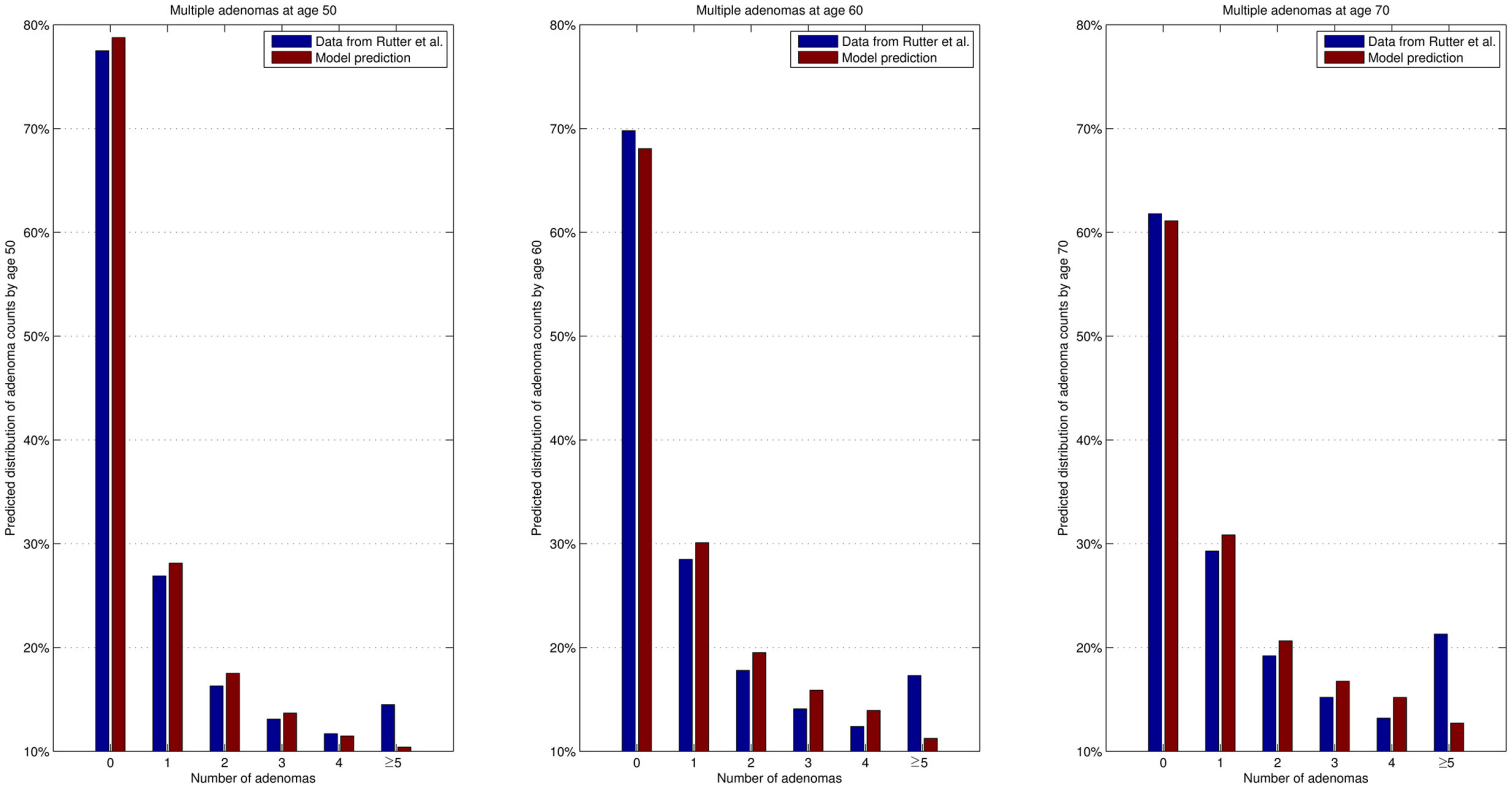
Numbers of all sorts of adenomas predicted by calibrated model at age 50, 60, 70 and the data given in [18].

**Fig 4:**
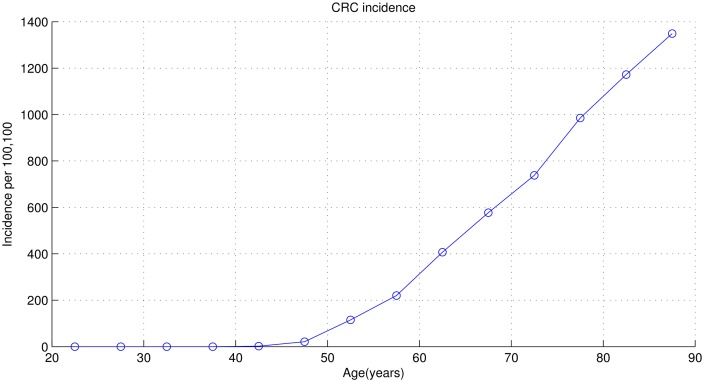
Numbers of CRC predicted by calibrated model at different ages.

Model calibrations are conducted step by step. Firstly, model parameters concerning adenoma pathway are calibrated with data given in [18]. Fig 3 shows the numbers of all sorts of adenomas predicted by our calibrated model and real data at age 50, 60 and 70. Then, we calibrate model parameters concerning cancer pathway with the total number of CRC at age 40-74 given in [11] and the trend of CRC incidence predicted in [6]. Fig 4 plots the final outcome of CRC incidence predicted by the calibrated model.

Tables 1 and 2 shows the model parameters that are derived from published literature or CanSPUC. Meanwhile, there are also some calibrated values.

**Table 1:** Initial parameter values used in the simulation.

Model parameters	Value	Resources
Sensitivity of colonoscopy		[4, 19]
Diminutive adenoma	0.74	
Small adenoma	0.87	
Large adenoma	0.979	
Cancer	0.99	
Sensitivity of FIT (%)		[4, 6]
Diminutive adenoma	0.0014	
Small adenoma	0.12	
Large adenoma	0.29	
Cancer	0.79	
Transition probability		[6, 9]
Basis risk of adenoma incidence (No adenoma to Diminutive adenoma)		
20–39	0.003	
40–49	0.007	
50–54	0.019	
55–59	0.022	
60–64	0.024	
65–69	0.028	
70–74	0.033	
75–90	0.035	
Standard deviation of incidence factor	1.7	
Diminutive to small adenoma	0.03	
Small to large adenoma	0.05	
Small adenoma to diminutive	0.195	
Large to small adenoma	0.05	
Large to small adenoma	0.05	
Advanced adenoma to preclinical early cancer	0.016	
Preclinical early cancer to clinical advanced cancer	0.3	
Preclinical early cancer to death	0.18	
Clinical early cancer to death	0.04	
Clinical advanced cancer to death	0.13	
Early cancer to curative resection	0.96	
Recurrence to preclinical early cancer after curative resection	0.1137	[9]
Recurrence to preclinical advanced cancer after curative resection	0.1439	[9]
Villosity(Tubular to tubulovillous/villous)		[6]
Diminutive adenoma	0.003	
Small adenoma	0.015	
Large adenoma	0.07	
Dysplasia(Low grade to high grade)		[6]
Diminutive adenoma	0.004	
Small adenoma	0.006	
Large adenoma	0.007	
Cost(¥)		[9, 11]
FIT	9	
Screening colonoscopy	290	
Pathology	150	
Colonoscopy with polypectomy	500	
Treatment costs of early cancer	4650	
Treatment costs of advanced cancer	26750	

**Table 2:** Initial parameter values used in the simulation.

Model parameters	Value	Resources
Annual utility of individual (*U*(*S*))		[17, 20]
No-adenoma or curative resection	1	
diminutive or small adenoma	0.955	
advanced adenoma	0.8	
early cancer	0.6	
advanced cancer	0.25	
death	0	

### Numerical Results

#### Baseline analysis

In this section, we compared the cost-effectiveness of all tested screening strategies with the parameters given in Tables 1 and 2 under baseline scenario where the initial screening age and ending screening age are set to 45 and 90, respectively. Moreover, compliance rates of FIT, first and follow-up colonoscopy are set to 45.37%, 37.32% and 50%, respectively. Table 3 shows all screening strategies with different intervals of FIT or colonoscopy, where ∞ means that at most one colonoscopy is conducted in individual’s lifetime.

**Table 3:** Screening strategies with different screening intervals.

	Strategy									
Interval	1	2	3	4	5	6	7	8	9	10
T_*f*_	2	2	2	2	1	1	1	1	1	1
T_*c*_(no adenoma)	3	10	∞	10	3	10	10	∞	10	∞
T_*c*_(1-2 diminutive or small adenomas)	3	3	3	10	3	3	6	3	10	6
T_*c*_(3-10 diminutive or small adenomas)	3	3	3	10	3	3	4	3	10	4
T_*c*_(advanced adenoma)	3	3	3	10	3	3	4	3	10	4
T_*c*_(early cancer)	3	3	3	10	3	3	2	3	10	2

Table 4 presents the cumulative costs, cumulative QALYs, ICERs and ICPCPs versus no screening for all tested screening strategies. As it is shown in Table 4, all screening strategies are cost-effective compared with no screening strategy. Moreover, strategy 10 is the most cost-effective strategy among all tested strategies, which recommends annual FIT plus observation-based colonoscopy screening.

**Table 4:** Accumulative costs, QALYs, ICERs and ICPCPs for all tested strategies.

Strategy	cumulative costs	cumulative QALYs	ICER vs. no screening	ICPCP vs. no screening
No screening	1656	23.23	-	-
1	1764.613	23.53998	351.8043	4804.9
2	1748.455	23.54231	297.5269	4063.3
3	1735.485	23.53002	266.5851	3558.5
4	1731.025	23.54088	242.6569	3277.5
5	1757.191	23.69258	219.378	3510.2
6	1737.082	23.69559	174.7741	2792.5
7	1733.311	23.69924	165.2389	2641.7
8	1709.928	23.67303	122.2666	1907
9	1711.523	23.69657	119.6285	1885.6
10	1708.104	23.68044	116.208	1823.3

Comparing strategy 5 with 1, 6 with 2, 8 with 3, and 9 with 4, we find that annual FIT is better than every two-year FIT in terms of ICER, ICPCP and cumulative costs. We also find that for adenoma-free individuals single colonoscopy is the most cost-effective strategy (comparing strategy 3 with 1 and 2, 8 with 5 and 6, 10 with 7). They are consistent with the findings in [9] and [11].

**Fig 5:**
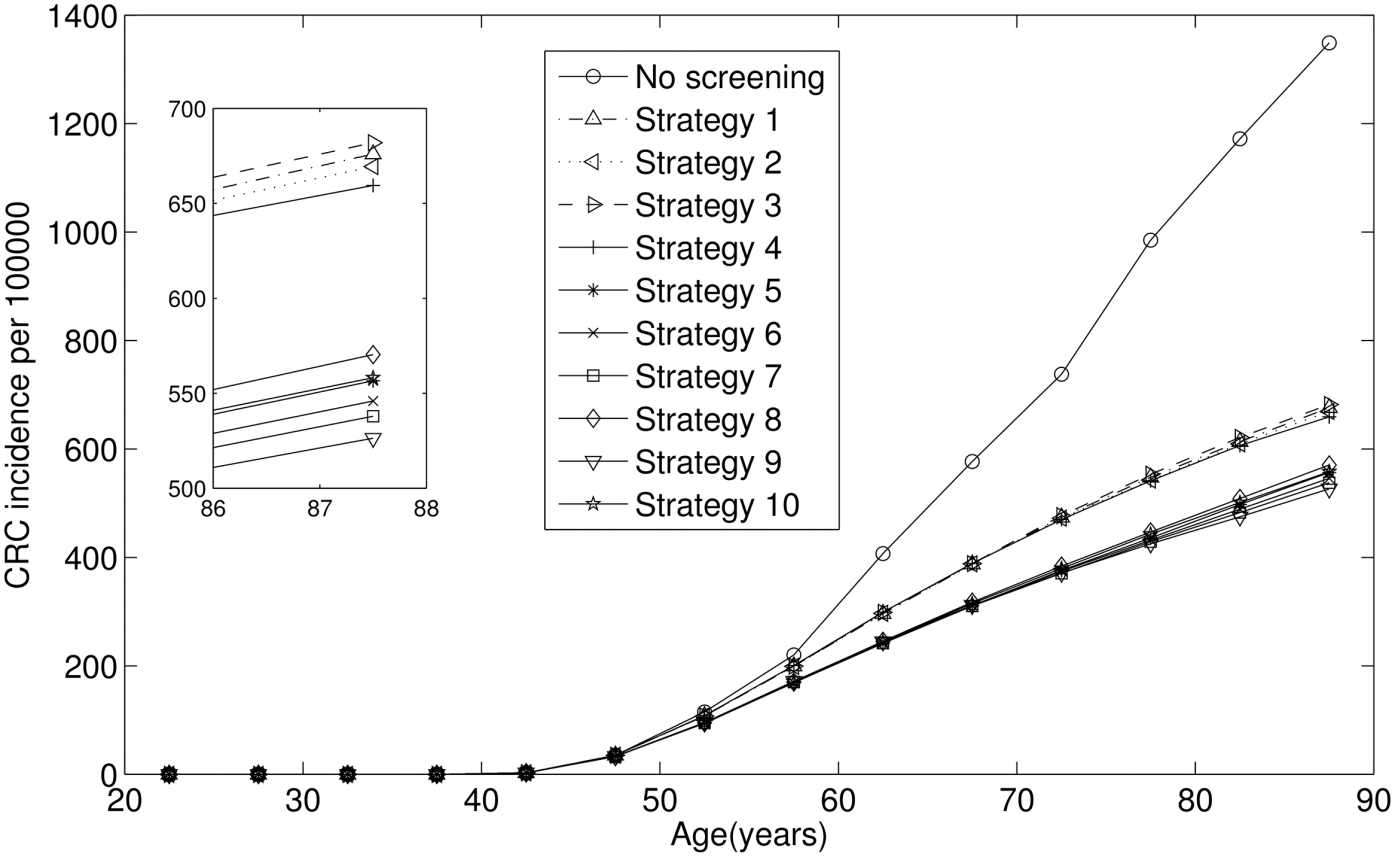
Numbers of CRC at different ages for all the tested screening strategies and no screening strategy.

Fig 5 plots the numbers of CRC at different ages for all the tested screening strategies and no screening strategy. Fig 5 shows that all screening strategies are effective considering the CRC number.

According to simulation results, strategy 4 (9) with every ten-year colonoscopy can reduce more CRC cases than strategy 1 (5) with every three-year colonoscopy (more frequently). Maybe the fact that advanced adenoma’s development needs ten years is an reasonable explanation. That is, every ten-year colonoscopy can just effectively detect and remove precancerous adenomas before they become cancerous.

#### Sensitivity analysis

**Fig 6:**
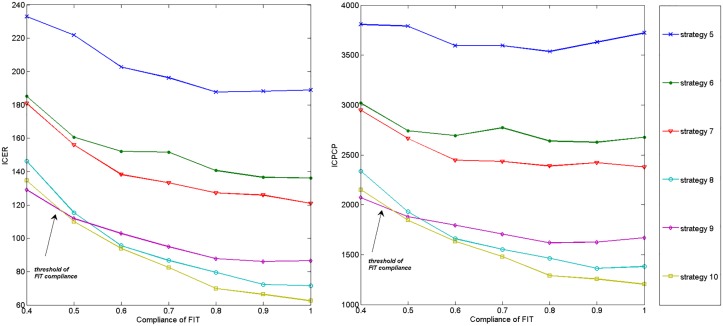
Impact of compliance of FIT on ICER and ICPCP vs. no screening.

**Fig 7:**
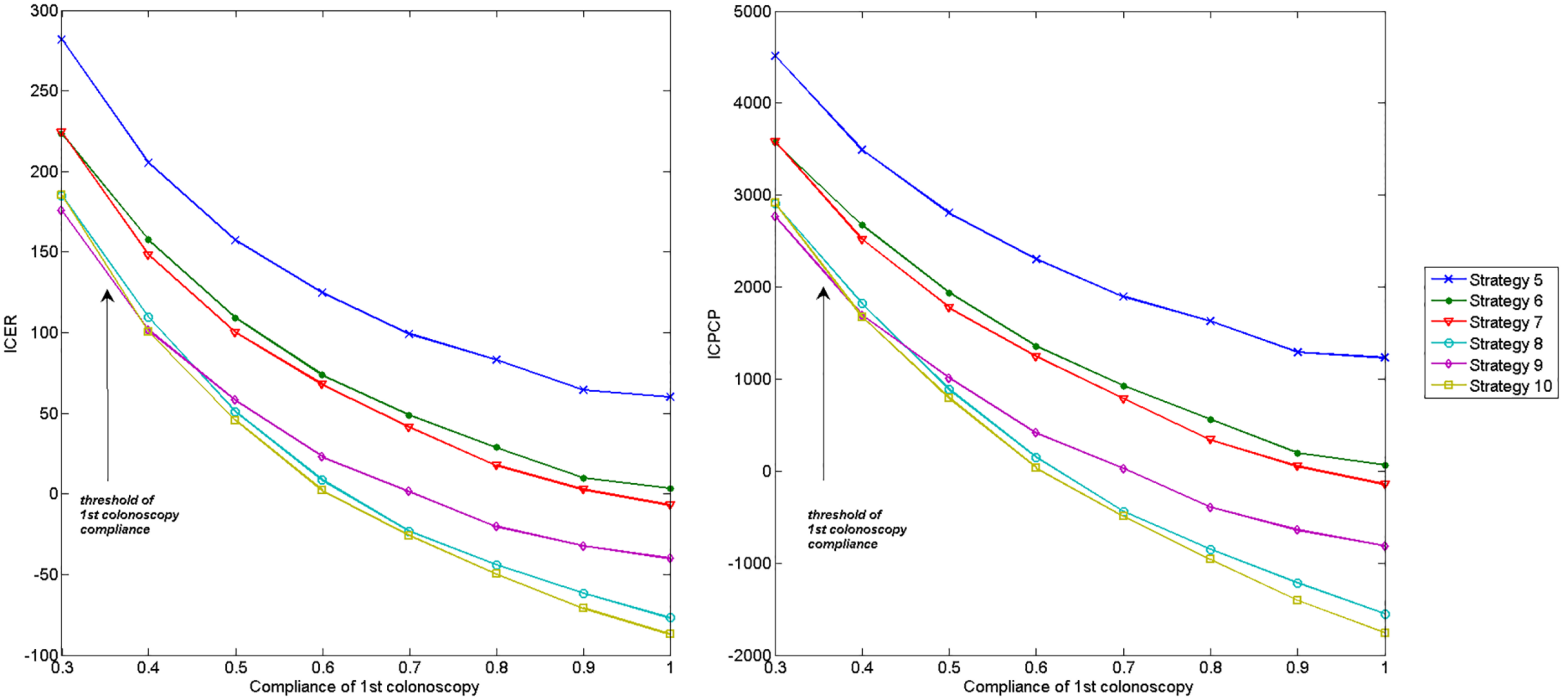
Impact of compliance of 1st colonoscopy on ICER and ICPCP vs. no screening.

**Fig 8:**
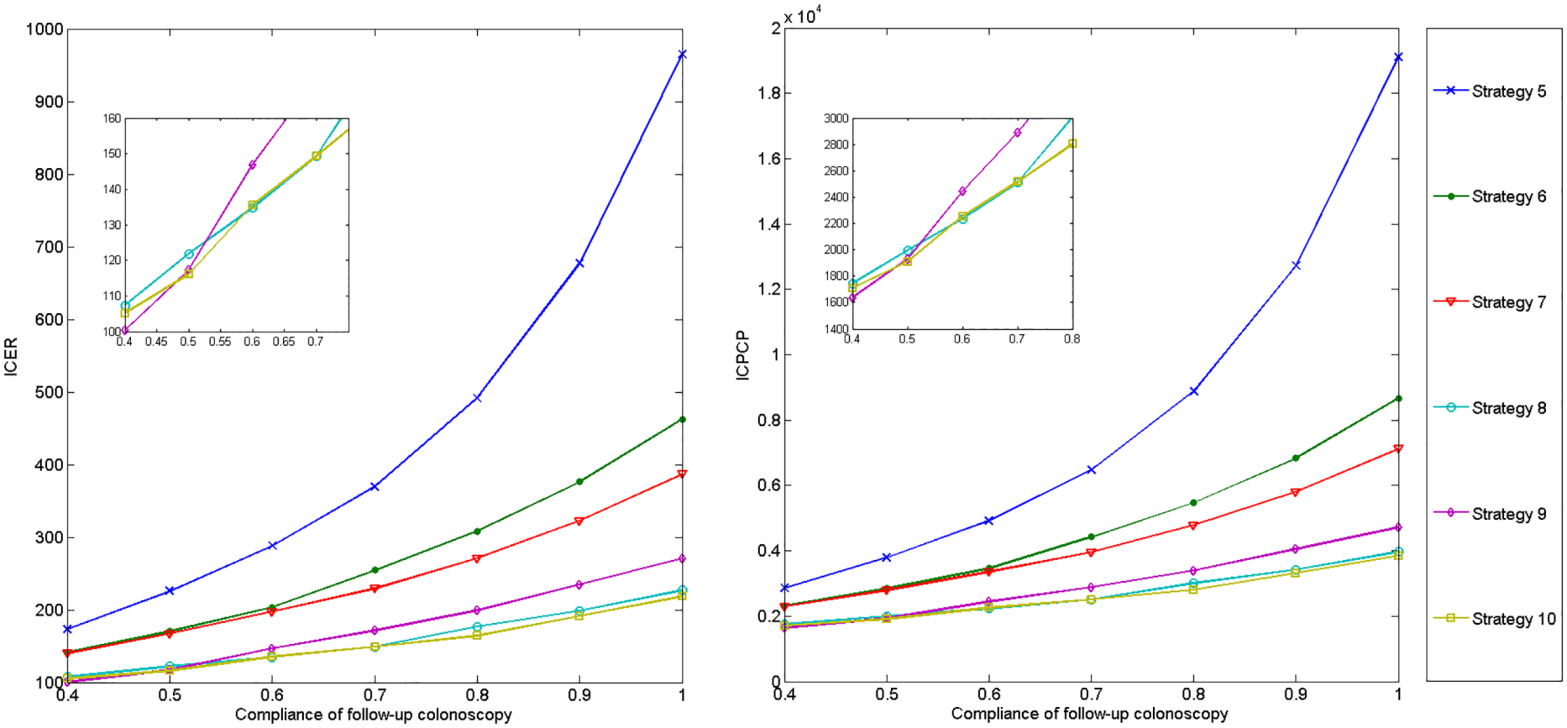
Impact of compliance of follow-up colonoscopy on ICER and ICPCP vs. no screening.

To explore the effect of compliance of FIT and colonoscopy, we first preformed one-way sensitivity analyses on the ICER and ICPCP vs. no screening. The compliance rates of FIT, 1st colonoscopy and follow-up colonoscopy are changed between 40% to 100%, 30% to 100%, and 50% to 100%, respectively. As annual FIT is obviously better than every two-year FIT, we only simulated with 6 strategies where annual FIT is adopted. Figs 6–8 plot corresponding results.

According to Figs 6 and 7, the ICER and ICPCP are decreased with the increase of compliance rates of FIT and 1st colonoscopy, except for strategy 5 and 6 in Fig 6. However, Fig 8 shows that both ICER and ICPCP are increased with rising compliance rate of follow-up colonoscopy.

We also find that some strategies are sensitive to changes in compliance rates, while some are less sensitive. There are threshold values at 40%∼50%, 30%∼40% and 40%∼50% for compliance rates of FIT, 1st and follow-up colonoscopy, below which strategy 9 is the most cost-effective, otherwise strategy 10 is the most cost-effective. Strategy 8 also performs better than strategy 9 after certain values, while the ranking order of other strategies remain unchanged with the increase of compliance rates.

We also performed two-way sensitivity analyses on the ICER vs. no screening. Here, only strategy 8, 9 and 10 are considered as they are obviously more cost-effective than others. The compliance rates of FIT, 1st colonoscopy and follow-up colonoscopy are changed between 40% to 100%, 30% to 100%, and 40% to 100%, respectively. Table 5.a, 5.b and 5.c plot corresponding results.

**Table 5:** Impact of compliance of FIT, 1st colonoscopy and follow-up colonoscopy on ICER: two-way sensitivity analysis.

Table 5.a FIT and 1st colonoscopy.				
Compliance of FIT	Compliance of 1st colonoscopy	Strategy 8	Strategy 9	Strategy 10
40%	30% ∼ 100%	197.7 ∼ -71.8	188.3 ∼ -42.8	196.5 ∼ -77.3
60%	30% ∼ 100%	160.2 ∼ -81.9	160.0 ∼ -40.0	151.9 ∼ -90.4
80%	30% ∼ 100%	139.4 ∼ -71.6	138.6 ∼ -18.0	128.7 ∼ -84.7
100%	30% ∼ 100%	127.5 ∼ -46.2	131.2 ∼ 8.4	116.4 ∼ -63.6
Table 5.b 1st and follow-up colonoscopy.				
Compliance of 1st colonoscopy	Compliance of follow-up colonoscopy	Strategy 8	Strategy 9	Strategy 10
30%	40% ∼ 100%	176.2 ∼ 281.2	154.3 ∼ 314.2	170.1 ∼ 274.3
50%	40% ∼ 100%	37.8 ∼ 157.0	34.0 ∼ 228.8	34.0 ∼ 158.0
70%	40% ∼ 100%	-32.0 ∼ 94.4	-29.8 ∼ 189.5	-37.7 ∼ 93.5
100%	40% ∼ 100%	-93.3 ∼ 41.1	-73.1 ∼ 172.3	-100.2 ∼ 43.3
Table 5.c FIT and follow-up colonoscopy.				
Compliance of follow-up colonoscopy	Compliance of FIT	Strategy 8	Strategy 9	Strategy 10
40%	40% ∼ 100%	123.6 ∼ 57.7	107.3 ∼ 65.5	120.8 ∼ 48.2
70%	40% ∼ 100%	165.8 ∼ 100.1	180.2 ∼ 149.9	161.6 ∼ 89.4
100%	40% ∼ 100%	235.9 ∼ 163.7	272.3 ∼ 266.3	234.4 ∼ 161.8

According to Table 5, we find that the results of two-way analysis are consistent with one-way analysis. That is, strategy 10 is most cost-effective in most circumstances; however, there are also threshold values below which strategy 9 or 8 is the most cost-effective.

## Discussion

In this paper, we present a model with detailed precancerous adenoma states to depict the natural history of CRC in average-risk Chinese and then evaluate various screening strategies using the calibrated model. Different from current researches, our natural history model considers the population’s heterogeneous risk of developing adenomas and age-specific state transition probability; and the FIT plus observation-based colonoscopy screening strategies are explicitly analyzed; and we present a new cost-effectiveness metric.

Numerical studies show that all tested screening strategies are cost-effective vs. no screening strategy. Moreover, an observation-based screening strategy is the most cost-effective, which recommends annual FIT, single colonoscopy for adenoma-free individuals and observation-based colonoscopy screening intervals for those with adenomas instead of fixed interval.

Compliance rates have a significantly influence on the cost-effectiveness of screening methods. Here, we explored all tested screening methods with a wide range of compliance rates. When compliance rates of FIT, 1st and follow-up colonoscopy are less than corresponding threshold values, strategy with every ten-year colonoscopy [9, 10] is the most cost-effective; otherwise, strategy with single colonoscopy for adenoma-free individuals and every three-year colonoscopy for those with adenoma is recommended except for the observation-based strategy [9, 11]. Thus, our findings give an explicit and complete instruction in CRC screening protocol in average-risk Chinese.

There are two limitations in this paper. First, the threshold values are obtained through simulations but not real data, which influence the choice of screening strategies. Second, our model does not incorporate the impact of screening results on compliance rates. In the future, we plan to solve these limitations by collecting more related data and models [21, 22].
